# Exploring Conversational and Physiological Aspects of Psychotherapy Talk

**DOI:** 10.3389/fpsyg.2020.591124

**Published:** 2020-11-05

**Authors:** Evrinomy Avdi, Chris Evans

**Affiliations:** ^1^Laboratory of Applied Psychology, School of Psychology, Aristotle University of Thessaloniki, Thessaloniki, Greece; ^2^Department of Psychology, The University of Sheffield, Sheffield, United Kingdom

**Keywords:** conversation analysis, psychotherapy process, autonomic arousal, nonverbal interaction, implicit domain, psychoanalytic psychotherapy

## Abstract

This study is part of a larger exploration of ‘talk and cure’ that combines the examination of talk-in-interaction with nonverbal displays and measurements of the client’s and therapist’s autonomic arousal during therapy sessions. A key assumption of the study is that psychotherapy entails processes of intersubjective meaning-making that occur across different modalities and take place in both verbal/explicit and nonverbal/implicit domains. A single session of a psychodynamic psychotherapy is analyzed with a focus on the expression and management of affect, with an aim to describe key interactive events that promote change in both semantic and procedural domains. The clinical dialog is analyzed discursively, with a focus on the conversational processes through which new meanings are jointly constructed and affective states shared; detailed attention is paid to nonverbal displays of affiliation and affect. Furthermore, we explore whether the interactional patterns implicated in joint meaning-making, as revealed by analyzing the therapeutic conversation, have correlates in the autonomic arousal of the two protagonists, as reflected in their heart rates. Conversation analysis has still untapped potential to illuminate interactional patterns that underlie the practice of psychotherapy. In this exploratory study we suggest that discursive analyses of talk-in-interaction can be enriched through detailed focus on nonverbal displays as well as measures of physiological arousal. Drawing upon the analysis, we suggest that bringing the methodological strengths of language-based analysis into fertile dialog with embodied quantitative data can help our explorations of what’s really going on in psychotherapy.

## Introduction

Although there is a proliferation of theories of psychotherapy, there is relatively little in-session research that explores in detail the processes through which change takes place as a session unfolds. In this exploratory single-case study, we examine in detail, and from different perspectives, one session of psychoanalytic face-to-face psychotherapy with an aim to describe therapeutic interaction on both explicit/conscious/verbal and implicit procedural levels (Stern et al., 1998). Our aim is to explore ways to expand our understanding of the interactional processes underpinning psychotherapy by studying the therapeutic conversation in conjunction with nonverbal displays (primarily of affiliation and disaffiliation) and psychophysiological measures of participants’ autonomic arousal during the session. Our methodological approach can be described as a ‘layered analysis’ (Avdi et al., 2020), as we examine the same interactional events on different levels and using different methods, and combine findings in an attempt to generate a multi-layered, clinically-informed description of therapy process. In line with the focus of this special issue, our broader aim is to better describe ‘what takes place’ in psychotherapy.

In this study, we approach psychotherapy as a relational process of intersubjective meaning-making; that is, a process through which client(s) and therapist jointly construct meaning, through multiple modalities of communication. Psychotherapy in this sense relies on a particular kind of conversation in a relational context that fosters the reconstruction of meaning and the reformulation of the client’s subjectivity (e.g., Avdi and Georgaca, 2018). Since its inception as the ‘talking cure’, language and meaning have been considered fundamental aspects of psychotherapy, and several discursive and conversation analytic (CA) studies have described different elements of the processes implicated in meaning construction in therapy (for reviews see Avdi and Georgaca, 2007; Peräkylä et al., 2008; Smoliak and Strong, 2018).

In recent years, however, there has been a growing recognition that psychological and social phenomena cannot be viewed in isolation from bodily processes, and researchers increasingly include affect and nonverbal, embodied aspects of communication in studies of human interaction (Cromby, 2012; Wetherell, 2015). The inclusion of affect and embodiment is arguably particularly pertinent in the study of psychotherapy, which entails affectively laden conversations about one’s self, life and relationships. Affect is intimately linked with meaning construction in psychotherapy and is an integral part of the work of therapy. Psychotherapy as an institutional practice promotes explicit discussion of the client’s affective experience and also examines the manifestation of affect in the session. In most psychotherapy schools, affective experience and expression are considered clinically relevant tasks that play an essential role in constructing new meanings and promoting therapeutic change. Furthermore, the experience, expression and processing (or working through) of affect -particularly negative affect- are considered key mechanisms of therapeutic action (e.g., Greenberg and Safran, 1989). Several important processes of therapy center on affective experience, such as the explicit naming of affect, mirroring and reflecting back the client’s affect, and the regulation of affect through the therapist’s ‘holding’ or containing presence (Slochower, 1991; Fonagy and Target, 1996).

Contemporary discursive theories assume that affect ‘permeates all utterances across all contexts’ (Besnier, 1990, p. 433). In this framework, embodied and affective processes are conceptualized as distinct, dynamic processes that are inscribed in discourse and therefore inseparable from it (Wetherell, 2015). In addition to explicitly referring to one’s emotions, affect is mostly conveyed implicitly through various discursive, linguistic, and communicative devices – such as prosody (pitch, tempo, volume of speech and pauses), intonation, lexical choice, syntax – many of which are context and culture dependent (Besnier, 1990). Generally, such work argues that the speaker’s affective state is usually alluded to through nonverbal means, rather than explicitly articulated.

Psychoanalytic theory has long recognized the centrality of embodied, affective experience for psychological functioning, our internal world, and our interactions with others. In psychoanalytic theory of change (e.g., Gabbard and Westen, 2003), a key mutative factor is ‘insight’: that is a process, comprising of cognitive and affective components, whereby unconscious motivation, wish, affect and other psychic elements become conscious. Insight operates within the declarative or conscious verbal domain and concerns knowledge that is explicit, readily brought into conscious awareness, and symbolically represented; in therapy it is promoted primarily through the therapist’s interpretations.

In addition to the role of insight, in psychoanalytic theory change is also mediated through the therapeutic relationship and analytic setting (Slochower, 1991). This process of change occurs on an implicit procedural or relational domain; it is non-conscious and represented non-symbolically in the form of *implicit relational knowledge*, i.e., knowledge about ways of ‘being with’ the other (Stern et al., 1998; Lyons-Ruth, 1999). Change of this sort is seen to rely on implicit, non-conscious relational exchanges between therapist and client, as they co-create a way of being with each other that produces qualitative shifts in procedural ‘knowing about relationships’ (e.g., Beebe and Lachmann, 2002, 2014; BCPSG, 2002, 2010, 2012). In contemporary psychoanalytic conceptualizations, shifts in implicit relational knowledge often take place following ‘moments of meeting’, that is moments of authentic person-to-person connection that are usually associated with heightened affect (Stern et al., 1998, p. 904). Stern et al. (1998) describe such moments as affectively ‘hot,’ unique moments of opportunity that, if seized, can bring about change. They represent moments where ‘the habitual framework -the known, familiar, intersubjective environment of thee therapist-patient relationship– has all of a sudden altered or risks alteration’ (1998, p. 911), often occurring when the therapeutic frame is challenged or broken. On a subjective level, they can be experienced as unfamiliar, unsettling or weird yet full of potential. Importantly for our purposes, such changes on an implicit level are closely associated with affect; these mutative exchanges implicate several affective processes, such as the mutual recognition and regulation of affective states, moments of affective understanding, and moments where this understanding is lost and then re-established in a process of self- and co-regulation of affect between interacting partners (Beebe and Lachmann, 2002; BCPSG, 2008). So, change in the implicit level takes place in an intersubjective context that is created through affective communication and mediated nonverbally; it includes several nonverbal behaviors such as vocal rhythms, gaze, orientation, intonations, posture, facial expression etc. (Stern et al., 1998).

These mechanisms of change, i.e., insight and shifts in implicit relational knowledge, are complementary, potentially mutually reinforcing, and often intertwined; however, they are distinct as they operate in different domains and through different change mechanisms (BCPSG, 2008). Drawing upon the above, in this study, we attend to nonverbal behaviors and autonomic arousal in addition to language, in an attempt to take into account both the explicit and implicit domains of psychotherapy process.

The therapeutic task, in line with these ideas, involves two key therapist activities: interpretations and ‘holding’ or containment (Slochower, 1991). Interpretations are a defining feature of psychoanalytic technique whose function is to make an unconscious phenomenon conscious (Gabbard and Westen, 2003). Interpretations invite further elaboration, reflection or emotional expression and aim to promote insight. In an interpretation the therapist introduces some additional meaning in what the client says, usually by providing links between different domains of experience, traditionally linking defense and anxiety in the context of past experience, current life and relationships, and the therapeutic relationship or transference (Malan, 1995). These meanings are thought to be unconscious, and possibly defended against, but evidenced within the client’s associations, transferential actions and affects. Interpretations often focus on the unconscious mechanisms employed in the service or resistance, i.e., defense mechanisms. The terms, ‘resistance’ and ‘defense’ have become utterly fixed in psychoanalytic theory but can be problematic as their use outside psychoanalytic theory can be quite pejorative. In psychoanalytic theory, resistance is a defense against insight, the active, unconscious opposition against recognizing aspects of one’s experience (a feeling, experience, memory, phantasy), when this knowledge is somehow unacceptable (Rangell, 1983). Psychoanalytic interpretations often focus on manifestations of resistance and explore the underlying affect/wish/idea/experience that is being defended against. Another type of interpretative activity involves transference interpretations that concern resistance in the context of the therapeutic relationship (Gabbard and Westen, 2003).

A metaphor commonly used to describe the non-interpretative aspect of psychoanalytic work is ‘holding,’ originally developed by Winnicott (1963), who drew parallels between psychotherapy and the parent-infant relationship. This term in the context of psychotherapy refers to the affective ‘holding’ provided by the psychoanalytic setting and attitude in the context of the therapeutic interaction. It is both a necessary backdrop to interpretative work and a curative factor in and of itself and is associated with the therapist’s reliably available and responsive presence (Slochower, 1991), which provides a regulatory function to clients’ affective arousal. Over the last decade there has been a shift from framing this as work done by the therapist to recognizing the bi-directional nature of interaction and to highlight the mutual co-regulation of affect in psychotherapy (e.g., Beebe and Lachmann, 2002; BCPSG, 2010, 2012). Interpretation and holding are not always easy to disentangle and are often mutually reinforcing.

Several studies using Conversation Analysis (CA) have examined the design, organization, trajectory, and function of interpretations in the context of psychoanalytic psychotherapy as well as clients’ responses to them (e.g., Vehviläinen, 2003; Peräkylä, 2004, 2008, 2010). These studies suggest that the preferred response to an interpretation is ‘an extended agreement’ (Bercelli et al., 2008), whereby the client provides further, usually autobiographical, material or elaborates, rather than simply confirming the idea or agreeing. A common way in which therapists encourage such elaboration, when it is not forthcoming, is by adding increments to the interpretation (e.g., Peräkylä, 2010).

Although the psychoanalytic concept of holding has not been studied explicitly by CA, several studies have examined the affective dimension of the clinical interaction, in line with the recognition of the importance of studying affect when examining social interaction (e.g., Voutilainen et al., 2010; Weiste and Peräkylä, 2014). In these studies, the expression and management of affect has been studied in relation to lexical and syntactic choices, pauses, as well as nonverbal displays such as prosody, gesture, and facial expression. For example, there is evidence that prosody plays an important role in creating meaning and regulating affect independently from the content of talk (e.g., Tomicic et al., 2014; Weiste and Peräkylä, 2014). Vocal characteristics such lower volume, slower rhythm, and softer intonation, as compared to surrounding speech, have been described as ‘soft prosody,’ and have been shown to be an important conversational resource in psychotherapy that can function to elicit emotional expression and to facilitate the emergence of new meanings (e.g., Weiste and Peräkylä, 2014; Kykyri et al., 2017). Other aspects of affiliative and empathic response include verbal continuers (e.g., ‘uh huh,’ ‘yeah’) and nods (Stivers, 2008; Voutilainen et al., 2019), affiliative (compassionate, caring) facial expression (Chovil, 1991; Peräkylä and Ruusuvuori, 2012), pauses (Levitt, 2001), and smiling.

Although psychoanalytic holding has not been studied using CA, the overlapping idea of the therapeutic alliance has, particularly through focus on the concepts of alignment and affiliation. Stivers (2008) suggested a distinction between alignment and affiliation, two separate functions of the listener’s response in the context of storytelling. Alignment concerns the activity of storytelling itself, and refers to cooperative actions that facilitate the conversational sequence. Affiliation refers to verbal and nonverbal actions that display acceptance and agreement with the teller’s affective stance, and as such is associated with empathy, rapport, reciprocity, engagement and interpersonal sensitivity (Lindström and Sorjonen, 2013). A few recent CA studies have used these concepts to examine the establishment, maintenance and repair of the therapeutic alliance in psychotherapy (e.g., Sutherland and Strong, 2011; Muntigl et al., 2013; Muntigl and Horvath, 2016).

Given that discursive research on the affective and nonverbal processes implicated in therapy is rather limited, and in order to contextualisz our study, in the next section we present literature from two related areas of research: nonverbal interaction in psychotherapy and interpersonal physiology in social interaction.

### Nonverbal Interaction in Psychotherapy

Human interaction is inherently multimodal, in the sense that it relies upon the intertwined cooperation of multiple channels of communication; these include the vocal/aural modality, i.e., speech and prosody, and the visuospatial modality, i.e., facial expression, body movement, gaze, gesture etc. (Stivers and Sidnell, 2005). These different modalities work together to construct meaning, through more or less coherent courses of action, and as such meaning is co-constituted across verbal and nonverbal modalities rather than merely constructed through talk (Cromby, 2012). Importantly for the context of psychotherapy, nonverbal aspects of communication and the manifestation of affect in talk are conveyed and processed primarily non-consciously, through an embodied form of knowledge (Besnier, 1990).

Given the multimodal nature of face-to-face dialog, the different modalities can work together, i.e., the same message being conveyed across verbal and nonverbal domains (for example tone of voice, facial expression and verbal content). However, these different modalities may at other times convey different messages. Such intermodal discrepancies can be either a communicative resource, as is the case in humor or sarcasm (e.g., Besnier, 1990), or potentially problematic for communication, especially if this incongruence goes unmarked. This has been described as creating a ‘double bind’ for the listener; in the infant development literature such intermodal discrepancies -e.g., simultaneous positive facial affect but negative vocal affect- have been described as affective communication errors, and are considered a risk factor for the development of disorganized attachment (e.g., Lyons-Ruth, 1999; Beebe et al., 2012). Importantly, there is some evidence that when such discrepancies occur between modalities it is generally the non-referential, i.e., nonverbal, signs tend to override other signs (Besnier, 1990).

Although limited, research on nonverbal processes (such as body orientation, postural sharing, smiling, nodding, and prosody) in psychotherapy suggests that nonverbal behavior is crucial for the therapeutic alliance and for the creation and communication of empathy (Hall et al., 1995; Knoblauch, 2000; Philippot et al., 2003). Much of research on nonverbal interaction has used the concept of *interpersonal coordination*, a term that refers to the degree to which the behaviors in an interaction are non-random, patterned or synchronized in both timing and form (Bernieri and Rosenthal, 1991). Two well-studied phenomena associated with interpersonal coordination are behavioral matching or mimicry (doing what the other is doing) and interactional synchrony (the intersubjective covariation of behavior or internal states in interacting partners, see e.g., Feldman, 2007). There is ample evidence that in social interactions we tend to match our behavior with that of our interaction partner on both verbal (e.g., tone, word choice, laughter, speech accent, syntax, and intonation) and nonverbal levels (posture, gesture, facial expression etc.). This occurs from very early in life and is considered an automatic, non-conscious process that is, however, regulated by top-down processes. Interpersonal coordination is associated with liking, affiliation, rapport, cooperation, self-other merging, perspective-taking, empathy, smoothness of interaction, and prosocial behaviors (Lakin and Chartrand, 2003; Chartrand and van Baaren, 2009). It is considered fundamental for the formation of social bonds, and it is assumed that it has evolved in order to communicate shared understanding and a sense of togetherness, and to help establish shared affectivity and empathy.

In the literature on psychotherapy ‘being in sync’ is thought to constitute a key component of rapport (Lakin and Chartrand, 2003) and has been associated with therapist responsiveness and the therapeutic alliance (Koole and Tschacher, 2016). In research on psychotherapy, for example, postural congruence and physical mirroring between client and therapist during sessions has been found to correlate with perceived empathy (Davids and Hadiks, 1994) and rapport (Raingruber, 2001), and movement synchrony has been shown to be positively associated with the therapeutic alliance, session quality and therapy outcome (Tickle-Degnen and Rosenthal, 1990; Ramseyer and Tschacher, 2006, 2011, 2014).

In a related line of inquiry, Bänninger-Huber and Widmer (1999) studied therapist’s nonverbal responses to client’s smiles in psychodynamic therapy and suggest that such nonverbal responses play an important role in regulating negative affect and constitute an important implicit component of the therapeutic alliance. Their findings suggest that an optimal degree of conflictive tension (associated with the therapist not responding to the client’s affiliative invitations), while maintaining relationship security is associated with therapy outcome (e.g., Bänninger-Huber and Widmer, 1999; Benecke et al., 2005). Similarly, research on ruptures and repairs of the therapeutic alliance (e.g., Safran and Muran, 2006) suggest that therapeutic interaction consists of periods of responsiveness interspersed with periods of mismatch and non-complementarity. In fact, there is evidence that psychological resilience, attachment security and therapeutic change are promoted through processes of rupture and repair in attunement and through mutual regulation, rather than simply by being in sync (e.g., Safran and Muran, 2006).

### Interpersonal Physiology and Psychotherapy Process

The autonomic nervous system (ANS) activation is closely associated with affective and cognitive processes, as well as other physical processes such as movement. For this reason, measures of ANS activation, such as electrodermal activity and heart rate, are considered correlates of affect and more specifically of the arousal component of affect. Affect is generally conceptualized in terms of two independent dimensions: valence (refers to its hedonic tone, positive/negative) and arousal (refers to the associated degree of bodily activation) (Posner et al., 2005). Autonomic activation is associated with the arousal component of affect, although the valence of affective experiences cannot be deduced from such measures. Indeed, it seems that different emotions such as anger, fear, happiness, and joy are all associated with increased physiological arousal, whereas sadness -and in particular sadness that is not accompanied by crying or anxiety- is associated with decreased arousal (Kreibig, 2010). Research on the physiology of social interaction generally and psychotherapy more specifically has a long history but is limited and rather fragmented (for recent reviews see Palumbo et al., 2016; Kleinbub, 2017). A review of this literature is beyond the scope of this paper, but findings from research on autonomic arousal in psychotherapy and other contexts that may be relevant to psychotherapy are briefly presented below.

There is some evidence that processes of self-construction, identity negotiation and positioning in social interaction are associated with increases in autonomic arousal, particularly in situations of threat to identity or blaming (Lyons and Cromby, 2010; Päivinen et al., 2016). In relation to the context of storytelling, Voutilainen et al. (2014) found that, when listening to stories characterized by ambivalence (i.e., where the storyteller had both a negative and positive stance toward the event narrated), the recipient showed increased autonomic arousal as compared to listening to ‘purely’ happy or sad stories. In a related study (Peräkylä et al., 2015), displays of affiliation by the recipient were associated with increased autonomic arousal in the recipient and decreased arousal for the teller. This finding was interpreted as reflecting a sharing of the ‘emotional load’ between interacting partners, whereby the listeners’ engagement regulated the teller’s physiological arousal (Peräkylä et al., 2015). This hypothesis was also explored in the context of psychoanalytic therapy with similar results: the therapists’ empathic displays were associated with increased arousal in the therapist and decreased arousal in the client; challenging, on the other hand, was associated with increases in the therapists’ arousal whilst challenging, and the clients’ arousal in the session as a whole (Voutilainen et al., 2018). On the other hand, in the context of couple therapy, clients’ autonomic arousal was found to increase when their words were mirrored by another speaker or when they were the topic of discussion, as well as sometimes during silent moments (Seikkula et al., 2015).

Findings from studies examining autonomic arousal in romantic couples suggest that lack of congruence between one’s feelings and behaviors as well as lack of emotional expressiveness are associated with increased arousal (Perrone et al., 2014). Similarly, actively suppressing emotional expression has been found to be associated with increased physiological arousal in both interacting partners (Butler et al., 2003). A similar argument was made by Marci and Riess (2005) in a single case study of psychodynamic therapy, where the client’s elevated autonomic arousal, in combination with her well-controlled demeanor, was interpreted as reflecting unexpressed affect. It seems that the suppression of affect may also be associated with increased autonomic arousal and in psychoanalytic terms this could be conceptualized as relating to intrapsychic conflict.

Another group of recent studies examining autonomic arousal in psychotherapy focus on physiological concordance or linkage, ‘the social coupling of two (or more) individuals in the here-and-now of a communication context that emerges alongside, and in addition to, their verbal exchanges’ (Tschacher and Meier, 2020, p. 558). Some early studies showed evidence for autonomic concordance between clients and therapists (e.g., DiMascio et al., 1957), a finding that has been explored further more recently (e.g., Marci et al., 2007; Villmann et al., 2008; Karvonen et al., 2015; Seikkula et al., 2015, 2018; Kodama et al., 2018; Tschacher and Meier, 2020). Findings from these studies are mixed; however, one finding that has been shown across several studies on psychotherapy sessions (as well as in studies of simulated sessions, e.g., Marci and Orr, 2006; Messina et al., 2013; Palmieri et al., 2018) is a correlation between ratings of empathy and the degree of physiological linkage between therapist and client. Despite some such relatively consistent findings, research on interpersonal physiology in psychotherapy is still in its infancy and is characterized by methodological and conceptual diversity which makes it difficult draw any definite overarching conclusions, other than that there is evidence of autonomic linkage between therapist and client during sessions (Kleinbub, 2017). Initial findings suggest that physiological linkage in interacting partners -both in the context of psychotherapy and other contexts- may be implicated in several different relational processes that are fundamental to the process of therapy, such as empathy and rapport, affect contagion and nonverbal, implicit communication of affect, the therapeutic alliance, and mutual affect regulation. These observations highlight the complexity of the therapeutic encounter and support the view that it is important to take into account nonverbal (and arguably non-conscious) aspects of the interaction when studying psychotherapy process.

### This Study

In this paper we adopt a case study approach and examine a single session of face-to-face psychoanalytic psychotherapy using conversation analysis, with an aim to track the process of therapy through one session. Although case studies are limited in their generalizability, they can illustrate important clinical concepts and techniques, help formulate hypotheses about clinical process, and can contribute to theory building. In this case study, we use a ‘layered’ analysis, in the sense that we examine the session on three different levels: conversation, nonverbal displays and autonomic arousal, and then combine these observations to produce a multi-layered description of the process of therapy.

## Method

### Materials and Methods

The material in this case study is drawn from a larger research project, conducted at the Aristotle University of Thessaloniki, Greece as part of broader study that aims to study the process of psychotherapy on multiple levels (Seikkula et al., 2015). It involves video-recording of sessions of face-to-face psychoanalytic psychotherapy conducted in a public, community mental health center that provides weekly psychoanalytic therapy. Ethics approval has been granted by the Center’s scientific council. To date, seven therapies, conducted by two experienced, female psychoanalytic therapists have been recorded, with a total of 137 sessions.

Clients are informed about the study at the intake interview and, if interested, are fully informed about the study by a graduate researcher. There are no specific inclusion criteria, as the study aims to explore routine clinical practice in naturalistic settings. All sessions are video-recorded and in specific sessions (at the start of therapy and then approximately every 6 months) both therapist and client wear heart-rate monitors to record their autonomic arousal during the session. Within 24 hours of these ‘measurement sessions,’ the researcher conducts Stimulated Recall interviews (Kagan et al., 1963) with the client and therapist separately. At the start of therapy and at the measurement sessions clients complete the CORE-OM (Evans et al., 2000) and the Working Alliance Inventory (Horvath and Greenberg, 1989). In this study, we do not refer to findings from the interviews.

The research material used in this case study consists of the video and detailed transcript of the session, including key nonverbal displays, and the autonomic arousal of each participant the duration of the session. With regards to autonomic arousal, both participants wore a small portable sensor (Firstbeat Bodyguard) that recorded their heart rate (HR) during the session. The sensors were synchronized using a Network Time Protocol (NTP) server to a resolution of 1 s and, at the start of each measurement session, the computer used to record the session was also synchronized with the same NTP server. Based on these measurements the Absolute Stress Vector (ASV), a second-by-second index that reflects sympathetic nervous system arousal, is calculated. The ASV is derived from the heart rate (HR), high frequency power, low frequency power and respiratory variables derived from heart rate variability (HRV): ‘ASV is high when heart rate is elevated, HRV is reduced, and respiration rate is low relative to HR and HRV’ (Kinnunen et al., 2006, p. 2). The ASV has been described as a new HRV-derived variable, which arguably shows resilience to heart rate artifacts and reflects sympathetic arousal more accurately than simple HR; it has been found to correlate with self-reports of stress (Myllymäki, 2006) and has recently been used in studies examining physiological arousal in psychotherapy^1^ (e.g., Seikkula et al., 2015; Kykyri et al., 2017).

### Methods of Analysis

The process of analysis was multi-layered and iterative. The session was transcribed verbatim and then key nonverbal displays were added to the transcript. Due to the nature of the analysis, which necessitates longer stretches of talk, the extracts are segmented into speakers’ turns, rather than lines as is more common in CA. In addition to transcribing verbal interaction, nonverbal aspects of the interaction were marked in the transcript, following the respective turn. These included displays of affiliation (facial expression, gaze, prosody, and smiling) and markers of tension and regulation of negative affect (adaptors). The transcription notation is shown in Table 1.

**TABLE 1:** Transcription notation.

**Symbol**	**Meaning**
(2)	Silence in seconds
(.)	Silence <0.2 s
.	Falling intonation at end of utterance
,	Continuing intonation at end of utterance
?	Rising intonation at end of utterance
(…)	Lines of extract omitted
°word°	Utterance spoken quietly
WORD	Utterance markedly loud
Word	Emphasis
.hhh	Audible inhalation
Hhh	Audible exhalation
	Stretch of talk slower
>*word*<	Stretch of talk rushed
heh	Laugh particles
wo:rd	Prolongation of sound
wor-	Truncated, cut-off speech
((cough)) ((sigh))	Audible non-speech sounds
[word]	Transcriber’s note
[	Starting point of overlapping talk
]	Endpoint of overlapping talk
((*looks away*))	Nonverbal behavior

For the analysis of talk, the session was initially segmented into topical episodes (TEs), i.e., periods of time during which a specific topic was discussed. Coding of the TEs was carried out independently by two researchers and any discrepancies were resolved through discussion. This initial thematic coding provides a broad-brush description of the main topics discussed in the session. Next, talk in each topical episode was examined through conversation analysis (CA) with a focus on talk about affect.

Conversation analytic is an approach to studying social interaction that draws upon ethnomethodology, a sociological approach developed in the 1970s that ‘seeks to explicate processes of inference upon which the everyday social order is based’ (Peräkylä et al., 2008, p. 12). CA examines the organization of interaction with a focus on the sequence of utterances, and over the last 15 years has been increasingly recognized as a powerful tool for psychotherapy research that can help study how affectively laden meanings can are transformed in and through the therapeutic interaction (for an introduction to CA in psychotherapy see Peräkylä et al., 2008). In this study, we draw upon and adapt the methods of CA to study one session of psychoanalytic psychotherapy with a focus on talk about affect as well as on the manifestation and management of affect in the session. More specifically, we initially examined talk-in-interaction on a macro-level, i.e., following the transformation of the meanings surrounding affect through the session, and subsequently focused on the ‘local level,’ by studying in detail specific interactional events of therapeutic work around the client’s affect, with a focus on the therapist’s verbal and nonverbal responses to the client’s expression of affect. We suggest that expanding the focus of analysis to longer stretches of talk than is common in CA allows us to trace the gradual transformation of meaning through the session, and to map the client’s changing responses to the therapist’s interventions across the full time-scale of the session. Finally, the contours of physiological arousal, as reflected in the ASV of participants, were examined in relation to the topics discussed and the interactional work carried out. ASV data were read from commercial software export noted above into R (version 4.0.2, R Core Team, 2020). The quantitative data analytic strategy was purely descriptive following the CA, reporting means and standard deviations for both protagonists across the whole session and within the TEs and using plots of the ASV to allow the reader to align the detailed analyses of the discourse both within the sequence of ASV changes across the full session, and, in “zoomed in” plots by extract.

### Case Description

The specific therapy was selected for further analysis in collaboration with the therapist. The therapy consisted of 30 sessions of face-to-face psychoanalytic therapy lasting 15 months, and the session discussed in this paper is session 19, which was the second measurement session. The therapist described the therapy as ‘stuck’ and said she struggled to make sense of the specific session.

The client, ‘Kate,’ is a white, heterosexual married woman in her late thirties, who came to therapy experiencing anxiety and depression, which she attributes to difficulties in her relationship with her father. Her father has serious health problems and Kate has been looking after him over the past few years, as his health gradually deteriorated. He is described as demanding, uncooperative, irritable, and at times verbally aggressive. This leads to many fights, and Kate feels intense guilt about her angry outbursts. She has two older brothers, who live away and are not involved in their father’s care. Her mother died several years earlier. During the course of therapy, Kate became pregnant and terminated therapy shortly before giving birth, despite the therapist’s encouragement for her to continue. The therapist is a senior female psychiatrist and psychoanalyst, in her mid-fifties, with over 25 years clinical experience.

## Findings

The session split into eight TEs, as shown in Table 2.

**TABLE 2:** Topical Episodes with time, duration, and autonomic arousal values for client and therapist.

**TE**	**Theme**	**Start**	**End**	**Duration (sec.)**	**Client ASV**		**Therapist ASV**	
					**Mean**	***SD***	**Mean**	***SD***
1	Initial problem construction: Kate’s guilt, sense of inner badness and fear about father’s death	9:02:41	9:07:40	299	219	45.3	100	13.1
2	Account of recent incident of father’s rejection	9:07:41	9:13:18	337	219	63.2	119	24.6
3	3 stories about difficult relationship with Father and his rejection	9:13:19	9:24:59	700	202	42.3	110	20.5
4	Father’s will and sense of injustice regarding her brothers	9:25:00	9:32:01	421	198	40.2	100	16.1
5	Frustration and anger with brothers	9:32:02	9:35:51	229	188	28.4	108	24.3
6	Interpretative work regarding anger, guilt and self-blame	9:35:52	9:40:56	304	166	38.4	121	16.3
7	Account of difficult relationship with father and sense that he doesn’t care	9:40:57	9:46:29	332	173	25.5	101	15.3
8	Interpretative work regarding anger, guilt and self-blame	9:46:30	9:49:52	202	164	24.1	130	28.3

We initially present a simple description of the trajectory of autonomic arousal through the session for both participants, followed by analysis of the session in terms of conversation and nonverbal interaction.

### Quantitative ASV Data

There are 2,863 s (47′16′′) in the session but 57 ASV values were missing for the therapist from 09:36:37 (2,041 s into the session) to 09:37:33 (2,097 s). These data -two sequences of numbers, one per person per second – are very simple beside the enormous complexity and elaboration of verbal and nonverbal communication data: the data from each person are purely one dimensional, there is no turn-taking, no overlaps, more accurately, every second is an overlap of two values. However, there are many ways to analyze such data and conventions about how best to simplify and highlight aspects of the data. Here, we have used plots against time and a violin plot to show distributions.

**FIGURE 1:**
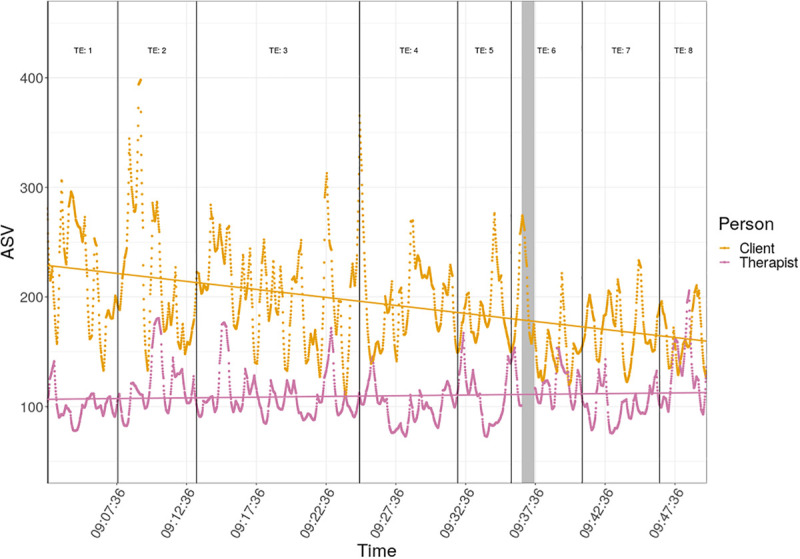
Absolute stress vector (ASV) and ASV linear trend against time for client and therapist. Vertical lines mark the boundaries between the numbered Topical Episodes (TEs). The gray vertical rectangle marks the missing therapist ASV values.

Figure 1 plots all the ASV against time across the whole session so as to map the data to the CA below.

This shows the marked changes in ASV over time for both participants; the client’s mean ASV (194.3) is higher than the therapist’s (109.7), so too for the variance (2,086 vs. 480.2). The client’s ASV declines across the session unlike that of the therapist. This is shown by the regression lines showing some fit to a simple linear relationship of ASV with time for the client.

**FIGURE 2:**
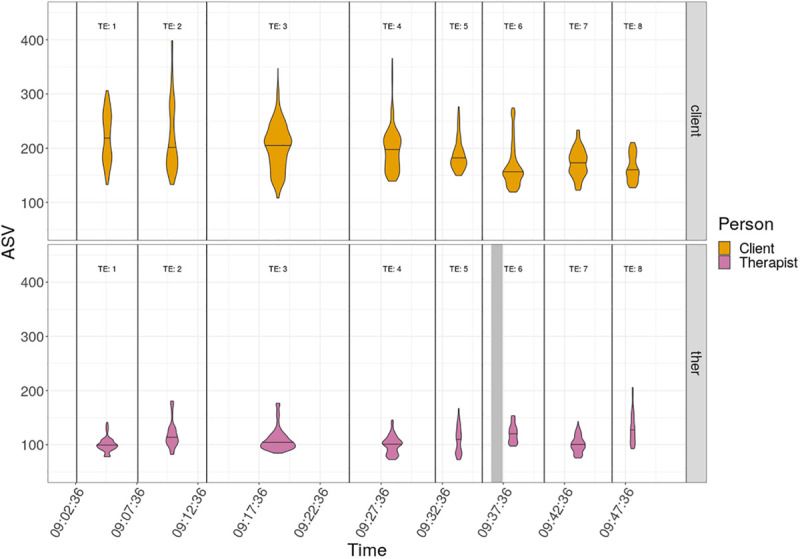
Violin plot of ASV of client and therapist by Topical Episodes. Separate violin plots of ASV are positioned on the midpoints of the TEs for the client and therapist. A violin plot gives a picture of the distribution of values. The areas of the “violins” are proportionate to the number of values hence the larger violins for the longer TEs and the larger violin for the client than the therapist for TE 6, which has the missing therapist ASV values. The violins stretch from the minimum to the maximum ASV value per person per TE and the horizontal waist lines on the violins mark the median scores for the person and TE. The widths of the violins show the actual distribution of values (which are marked on the *y*-axis).

Figure 2 is a violin plot organized by the TEs. This removes the sequence of the ASV changes within each TE so, instead of the jagged ups and downs, the distribution of values within the TE can be more readily observed. It can be seen that the means and variances vary quite markedly between TEs, markedly more so for the client than for the therapist. It can also be seen that, as well as the differences in means, ranges and variances, there are clear differences in the distributions of values between TEs: some are bimodal, i.e., with two distinct most frequent values, e.g., client TE 7, though most are unimodal; some show strong “skew” with a long thin distribution of high ASV above the median, very different from the short wide distribution below the mean, e.g., Therapist TE 3.

### Analysis of Conversation and Nonverbal Interaction in the Session

In terms of content, the key theme of this session, which is a central issue throughout the therapy, concerns Kate’s difficult feelings toward her father; she becomes angry with him easily and behaves abruptly toward him, and is then riddled with guilt. Much of the discussion through the session is oriented toward resolving the affectively charged ‘puzzle’ that Kate introduces in the first topical episode: her recurring, persistent, crippling guilt, and sense of internal badness. In the analysis that follows we examine interactional work around affect with a focus on the therapist’s verbal and nonverbal responses to Kate’s affective expression and the ensuing, gradual shifts in affective experience and meaning construction.

#### Initial Phase: Shifting Affect From Anger to Longing

In the initial phase of the session, which includes TEs 1–3 and lasts approximately half of the session time, Kate introduces the problem and then narrates several incidents concerning her relationship with her father that illustrate his ‘difficult character’ and his rejecting behavior toward her. The conversation during this phase is asymmetrical, in the sense that Kate speaks in long stretches of talk, in an animated tone, and expresses her frustration with her father both verbally and nonverbally. The therapist, speaks very little in this initial phase; in the first 25 min of the session, she only utters seven turns, out of which six are brief formulations that focus on Kate’s affect and one is a question inviting reflection, in response to Kate’s initial turn. This is characteristic of psychoanalytic practice in which space is given for the clients’ free association to develop in order for the unconscious associations to gradually manifest. Below, we present two extracts from this initial part of the session to illustrate the interactional processes implicated in managing affect.

This session takes place after a one-week break and starts with Kate providing an account of a difficult time she had during the intervening fortnight. She reports how one day she started to cry and was unable to stop, repeating the phrase ‘I am a bad person,’ filled with guilt about having shouted at her father during one of their arguments. She completes this initial problem description in a reflective manner, wondering what has changed: she used to feel justified in her anger toward her father, but this has recently changed. Following Kate’s opening turn, the therapist joins in her account and invites exploration of the factors that may lie behind her increased guilt. Kate tentatively suggests that perhaps her guilt is associated with underlying fear and sadness about the prospect of her father’s death. In this initial construction, the problem is defined as relating to Kate’s strong negative feelings, which are represented as outside her control and understanding; as such the agenda for the session is set to help solve the ‘puzzle’ of Kate’s intense guilt and anger.

**FIGURE 3:**
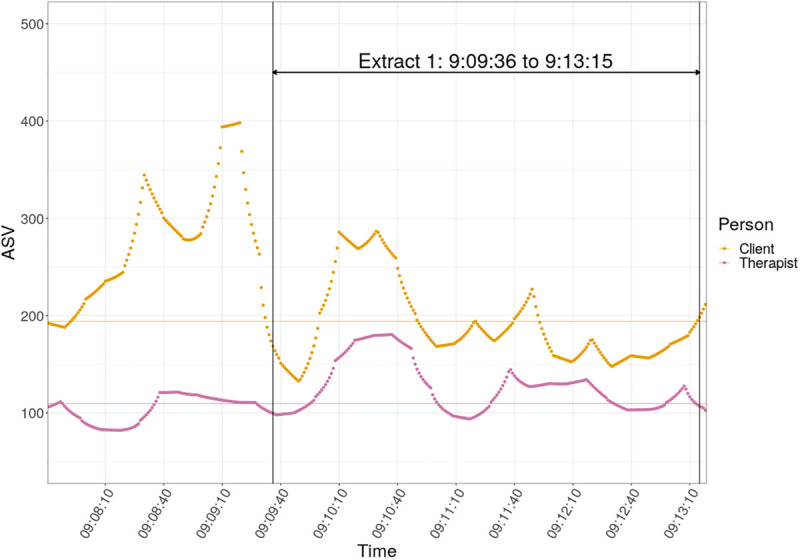
Absolute stress vector against time for client and therapist, TE2.

Following the opening interaction described above, Kate narrates a recent incident with her father and she concludes with the evaluation that it upset her: she was driving her father to a regular hospital appointment and had arranged to stop briefly and meet a friend on the way there. This friend complimented her on her creativity, praising something she had recently made; her father did not acknowledge the compliment but rather complained about the delay in getting to the hospital (the whole narrative with its introduction and evaluation lasts from 9:07:40 to 9:10:51). Extract 1 below follows the narration of this incident and the ASV of the protagonists through the extract is shown in Figure 3.

**EXTRACT 1:** From 9:09:36 to 9:13:15.

1a	K	(3) .hhhh and this upset me, once more, [although it is] something that I know, it has happened before in be:tter and worse ways (.)
		*((K gestures and then touches her face))*
1b		And then I said to him, dad, I say, what do you want me to do? To die completely? 100 percent? like (.) > not spend any time on my own things? on the things that I have to do? all the time<, most of my time I do things for you
		(…)
1c		and I told him (2) if it was anyone else, I say, > and somebody said something like that, like, they would smile, they would smile from ear to ear, if somebody said something good about their child, and you, like, the only thing you care about is that we won’t get there [to the hospital] at quarter to as you wanted?< and even then (1) .hhh ((sighs)) he didn’t say anything
1d		and then (.) I was thinking about (3) my mother ((*K bites lips*)) how she was the exact opposite of that, like, m:y mum (.) like, I would make >a small ball out of plasticine< and she would act as if, like, I: had received a pri:ze in nuclear physics (1) oh, look at what my child made (.) and it could be nothing, for her (.) in her eyes it was the: greatest thing in the world
		((*T neutral facial expression*))
2	Th	((coughs)) °You miss that a lot°
		((*T empathic facial expression*))
3	K	I do miss it, on the other hand I was thinking that that >perhaps my mother EXAGGERATED because she saw that nothing like this was coming from my father< ((*gestures*)) (…) perhaps she exaggerated because (.) my father showed (.) no positive response to anything like that
4	Th	So, he disappointed you (0.5) °in the past too°<°°your father, [not just now°°>
		((*T empathic facial expression*))
5	K	[((sighs)) ((*K bites her lips*)) (2) I don’t have a very clear memory but (.) I don’t remembe:r my father saying well done for anything (.) of course he was away a lot (.) I don’t know if I don’t remember it because I have repressed it (.) >I don’t know if I don’t remember it because this over-< because my mothe:r’s exaggerated joy was enough for me (.) I don’t know

At the start of turn 1, in completing her storytelling Kate evaluates it as part of a pattern (‘it happened before’) and as affectively relevant (it ‘upset me’). She then (1b) begins to ‘replay’ the dialog with her father, addressing him as if he were present. Enacting part of a dialog is a powerful way to convey affect in conversation and possibly induce it in the listener (Besnier, 1990). In discursive research, vivid descriptions are considered a rhetorical strategy of factualization, that is as a way of rendering an account plausible as accurately depicting facts. In this way, the possibility of one’s account being assessed as biased by the listener is minimized (Edwards and Potter, 1992). Similar ‘enactments’ are a common feature in Kate’s talk in this session. From a psychoanalytic perspective, this turn design could be seen as a (non-conscious) way of managing guilt: Kate talks as if she *expects* the therapist to doubt her version of events or assume that she is somehow at fault, and so her account is structured in such a way as to convince that it is a true and accurate record of what actually happened. During this narration, Kate’s talk becomes louder and more animated; the therapist, however, displays few signs of engagement and alignment with Kate’s story: she has a neutral facial expression, looks away from Kate much of the time, and does not provide any verbal continuers.

In terms of autonomic arousal, as can be seen in Figure 3, Kate’s arousal reaches its highest value in the session as she narrates the incident with her father described above (9:08:22 – 9:10:51), and her ASV remains elevated throughout the interaction presented in Extract 1. The therapist’s arousal also rises significantly during Kate’s storytelling, and peaks about 40 s after Kate’s highest peak, during the evaluation presented in turn 1. Furthermore, as can be seen in Figure 3, for a period of about 50 s during this interaction (9:10:09 – 9:11:00) both participants’ ASV is elevated. It is interesting to note that, although the therapist displays few nonverbal signs of ‘being with’ Kate during this narration, her autonomic arousal peaks soon after Kate’s highest arousal point, in what could be considered an indication of physiological linkage or, in clinical terms, embodied responsiveness. Following this, and for the remainder of Extract 1, described below, both participants’ ASV is not particularly elevated.

In response to Kate’s turn, the therapist makes a brief formulation (turn 2) that selectively focuses on Kate’s affect, thus shifting focus from the description of events to affective experience. Formulations are utterances that show understanding of the previous speaker’s turn by proposing a version of it; at the same time, they often subtly change what has been said through selection, deletion and transformation (Antaki et al., 2005). Formulations are used extensively in psychotherapy and serve several different functions that promote the work of therapy, such as displaying understanding, transforming clients’ complaints into psychological difficulties that can be addressed through therapy, managing the progress of interaction etc. (Antaki, 2008). Weiste and Peräkylä (2013) suggested that three types of formulation tend to be used in psychodynamic therapy. *Highlighting formulations* show understanding of the client’s turn, and selectively highlight its clinically relevant aspects, which often relate to affect. In *rephrasing formulations*, that usually concern the client’s subjective experience, the therapist proposes his or her version of the client’s subjective experience by renaming it, and in this way invites self-reflection and further emotional expression. *Relocating formulations* propose links between the experiences described in the client’s turn and experiences that took place at other times (usually childhood) or in other (relational) contexts.

Through this brief formulation (turn 2), the therapist sidesteps reference to the father’s behavior or to Kate’s own anger and focuses instead on Kate’s subjective experience of lack (‘you miss that’). This is designed as an extension of Kate’s talk, in the sense that it is spoken from within Kate’s perspective, but does not focus on affects that are expressed (i.e., frustration and anger) but rather on lack and longing: Kate has not mentioned these affects but the therapist deduces them from Kate’s associations. In this way, the therapist names Kate’s not-yet expressed feelings of missing parental approval. This shift from facts to feelings and from anger to longing is also facilitated nonverbally; as she speaks, the therapist uses a very low volume voice, which is intimate and soothing, looks at Kate with a concerned facial expression, and smiles slightly.

In response (turn 3), Kate provides a minimal confirmation followed by a disjunction (‘on the other hand’) and shifts focus again on her mother, wondering whether her mother was overly encouraging so as to counteract her father’s lack of approval. From a psychoanalytic perspective this shift would be considered an example of resistance; Kate momentarily gets in touch with her feelings of missing parental approval but very quickly moves away from them, as, presumably, they are too painful. In her next turn (turn 4), the therapist does not respond to Kate’s reference to her mother but steers the conversation back to the clinically relevant issue of Kate’s disappointment in her relationship with her father. Furthermore, she adds another increment in the formulation, suggesting that disappointment is a long-standing issue in Kate’s relationship with her father. In this way, the therapist invites Kate to talk about the past; this is another important aspect of psychoanalytic technique, where current difficulties are explored in relation to past experiences. In addition, the therapist replaces the rather vague construction ‘you miss that’ (turn 2) to ‘he disappointed you,’ thus naming Kate’s feelings more specifically and placing them in a relational context. In terms of meaning construction, the therapist introduces the idea that Kate feels disappointed in her relationship with her father and has felt so since childhood, although Kate has not made any such reference. Again, the therapist delivers this formulation in very low volume voice, with a soothing and affiliative tone, and displays a compassionate facial expression.

**FIGURE 4:**
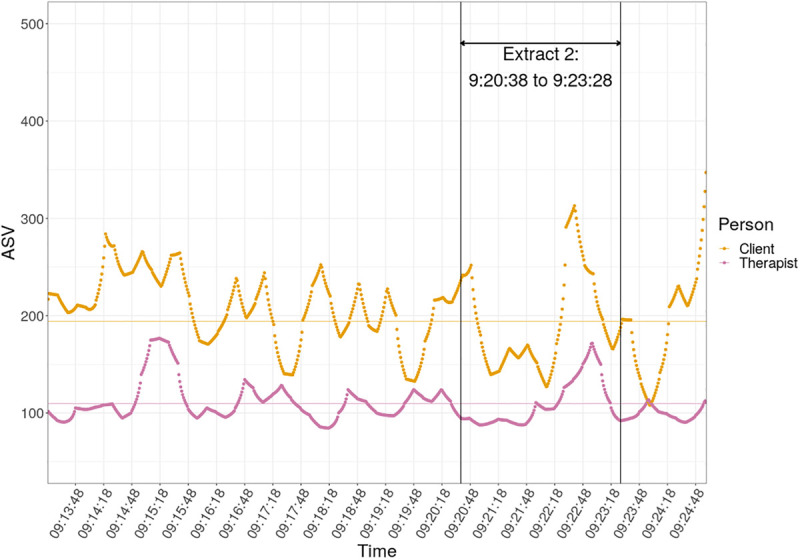
Absolute stress vector against time for client and therapist, TE3.

In response to this repeated invitation to talk about her disappointment, Kate hesitates and then disconfirms the therapist’s suggestion (turn 5): she cannot remember her father disappointing her and provides several explanations for this. This opposition to experiencing disappointment would be considered another manifestation of resistance from a psychoanalytic perspective. This time, the therapist remains silent and Kate recounts three further incidents from her recent past, over an 8-min stretch of uninterrupted talk. It is interesting to note that, although Kate opposed the suggestion that her father disappoints her, the stories she spontaneously narrates are examples of her father’s lack of recognition and approval; as such they can be considered elaborations in response to the therapist’s invitation to talk about her disappointment. The first story concerns an incident that occurred several years earlier. At that time, her father still lived on his own and Kate used to visit him regularly. On one such visit, she expressed her wish to rest but her father wanted her to cook something for him; he became very angry when she delayed preparing his meal and screamed at her to leave his house. She left the house, drove to the cemetery, and sat by her mother’s grave, crying for several hours. In terms of affect, there is a mismatch between the sad content of the story and the angry affective tone of the storytelling; in psychoanalytic terms, this mismatch could be conceptualized as an indication of internal conflict and defense (anger as defense against sadness). In terms of autonomic arousal, as can be seen in Figure 4, both participants’ ASV is elevated during the narration of this story (from 9:14:53 to 9:15:40).

The next extract starts with the therapist’s response to Kate’s storytelling described above.

Extract 2 illustrates Kate’s gradual acknowledgment of her sadness and longing for her father’s recognition and approval. In response to Kate’s storytelling, the therapist repeats, in a soothing and affiliative tone, her suggestion that she is disappointed (turn 6), thus inviting and validating Kate’s hidden feeling both verbally and nonverbally (Voutilainen et al., 2014). Kate confirms this minimally (turn 7), but then blames herself for feeling this way; again this would be considered a sign of resistance, as it shifts focus away from the painful feeling to frustration and self-blame. This time, however, the therapist persists (turns 8 and 10) and challenges, albeit in an affiliative manner, Kate’s reporting of her own experience. This leads to an extensive agreement by Kate (turn 11), as she talks about her longing for her father’s recognition and expresses her sadness that this is not forthcoming; the therapist validates these feelings with a brief formulation that highlights her wish (turn 12).

**EXTRACT 2:** From 9:20:38 to 9:23:28.

6	T:	you feel very disappointed
		((*gentle tone of voice and empathic facial expression*))
7	K:	I am disappointed, on the other hand I think (.) that it’s my fault because >he has said it once, twice, five times, ten times, a hundred<, eh ENOUGH, I should not keep asking for this ((*shrugs*)) approval
		((*T empathic facial expression*))
8	T:	((*shrugs*)) ye:s, but (.)°this is not how things are inside you°
		((*K wrings her hands and shifts in her seat*))
9	K:	(2) ((sighs)) ((*wrings hands*))
10	T:	inside you, this is something that you need, °and you feel disappointed°
11	K:	(6.5) ((sighs)) (.) that is the truth Hhh ((*C wrings her hands*)) (…) it’s NOT THAT I have, like (0.5) that I want my father >telling me every day< well done my child, thank you (…) I don’t expect that ((*K touches her face*)), just a little (1.5) some recognition for wha:t (1) for what I do, on a personal level ((*purses lips*)) (…) but why is it so difficult for him? (1) to show me, to show me in some way that yes ((tch)) I recognize tha:t (.) I see that you are trying, that’s all
12	T:	°°that is what you’d want °°
		((*empathic facial expression*))

In terms of autonomic arousal, as can be seen in Figure 4, both participants show elevated ASV during the affectively charged interaction presented in Extract 2. Kate’s ASV peaks when she expresses her wish for her father’ recognition and approval (turn 11) and remains elevated until the end of the extract (9:22:33 – 9:23:38). The therapist’s arousal peaks about 20 s after Kate’s highest ASV. As such, it seems that as Kate gets in touch with her longing for her father’s approval, the therapist’s autonomic arousal also rises, in what could be considered an indication of physiological linkage between participants and affective responsiveness.

In sum, the extracts described above entail interactional patterns that are quite typical in this session: Kate narrates several incidents that focus on her father’s rejecting behavior that angers her. She recounts these incidents in vivid detail but makes only vague reference to her subjective experience. The therapist responds with brief formulations that focus on feelings that lie ‘behind’ her anger; in psychoanalytic terms these feelings would be considered defended against, i.e., unconscious. Kate opposes these formulations and the therapist remains silent allowing Kate’s associations to emerge. The stories that Kate narrates are arguably elaborations in response to the therapist’s formulation, although they are not marked as such. In psychoanalytic terms, this delayed elaboration could be considered an example of free association, where the client on the one hand resists, whilst on another level responds to the therapist’s intervention.

In terms of the characteristics of the conversation, in the initial phase of the session, talk is distributed asymmetrically between participants; Kate has quantitative and semantic dominance, in the sense that she talks most, often in long stretches of talk, and introduces the topics of discussion in each episode. The therapist is silent much of the time and her talk is in the form of brief formulations that refer to Kate’s hidden affect, as described above. With regards to nonverbal aspects of the interaction, there is a marked difference in the prosodic features of the participants’ talk throughout this initial phase. The client speaks in fast tempo, in a loud, modulated and fairly high-pitched voice; these prosodic characteristics are often associated with physiological and emotional arousal (Soma et al., 2020). The therapist, on the other hand, speaks very quietly, in a low volume, slow rhythm, and low pitch voice. This marked lack of prosodic matching can be seen as a nonverbal, implicit process of self- and mutual affect regulation on the therapist’s part, which functions both to soothe and to invite the expression of painful feelings. In addition, the therapist alternates between misalignment and affiliation toward the client’s narrative, both of which arguably promote the work of therapy. When she speaks, the therapist displays many nonverbal signs of affiliation and empathy, thus fostering a sense of safety and inviting deepened affective experience. On the other hand, she shows few signs of engagement and affiliation when listening to Kate’s storytelling. Although non-affiliative responses to the client’s narration are considered non-preferred (Stivers, 2008) and arguably impact negatively the therapeutic alliance (Safran and Muran, 2006), from a psychoanalytic perspective they can be seen to promote the work of therapy by maintaining (unconscious) conflict, which eventually leads to emotional expression and self-reflection.

#### Latter Part of the Session: Working With Resistance and Managing Self-Blame

In the latter part of the session (TEs 6–8), the therapist shifts to more active interpretative work in the face of continuing resistance on Kate’s part. This part differs markedly from the initial half in terms of conversational characteristics. The therapist talks significantly more; her utterances are longer, and the majority of her turns are designed as rephrasing formulations. In addition, the therapist responds more promptly to Kate’s disagreements and actively interprets her resistance. In this part of the session Kate assumes too much agency for the difficulties in her relationship with her father and oscillates between anger and self-blame. This is interpreted by the therapist as a manifestation of resistance: Kate gets angry and blames herself in order to avoid experiencing disappointment. A fairly long extract from this part of the session is presented below, with an aim to illustrate this aspect of psychoanalytic work and explore its affective and embodied dimensions.

The interaction described takes place at the start of TE 6. Extract 3 is presented in three consecutive segments and is used to illustrate how the therapist gradually builds a psychoanalytic interpretation that links various aspects of Kate’s talk and provides an explanation for Kate’s intense guilt.

**EXTRACT 3A:** From 9:35:52 to 9:36:54.

1	T:	Err yes, but ((clears throat)) (2) °you get very angry with your father° ((coughs)) and the angrier you get (2) °°the more guilty you feel°°
		((*T empathic facial expression*))
2	K:	(2) Hhhhh
3	T:	So, while, it angers you that he does not recognize the things you do, in many different ways, the fact that, you, <there are many things that you cannot not do and so you leave your own life behind (.) eh, all this> ((coughs)) I am sorry (.) all this though (.) is something (.) that makes you °very angry° (.) and then, the angrier it makes you
		((*K looks away, wring her hands, sad facial expression*))
4	K:	I feel guilt
5	T:	the more you feel that you are doing something bad
		((*K wrings her hands, looks down, sad facial expression, bites her lips*))

In the first part of this interaction (Extract 3A), the therapist introduces a topic shift. Kate has been talking about her frustration with her brothers over the previous two TEs (4 and 5) and the therapist shifts focus abruptly on Kate’s feelings of anger and guilt in relation to her father. Although this sudden shift is misaligned with Kate’s previous turn, it is designed as if it were a continuation of her talk and is spoken in a gentle and low volume voice, accompanied by a concerned facial expression. Kate does not respond verbally but sighs (turn 2). In the next part of the formulation (turn 3) the therapist refers for the first time in the session to apparent facts (‘there are many things … life behind’) rather than Kate’s subjective experience. This externalizing shift from feelings to facts functions to validate Kate’s description of events as accurate, and by implication her reactions as justified. In this construction Kate is represented as having no choice but to do all the things she does for her father, which result in her leaving ‘her own life behind’ (a phrase used by Kate earlier in the session). In this way, her anger is an understandable and justified response to the situation she finds herself in. Throughout the therapist’s turns, Kate does not respond verbally but displays markers of negative affect. Kate completes the therapist’s turn (turn 4), thus jointly constructing an account that explains her guilt as resulting from her justifiable anger. The therapist, on the other hand, suggests a more experience-near description (turn 5) (‘you feel you are doing something bad’), using the words Kate introduced in the very beginning of the session (‘I am a bad person’). Using the client’s words from different parts of the session is one way in which therapists weave disparate experiences and affects into a coherent story, thus creating links between meanings that remained disjointed in the client’s talk.

Following this joint construction, however, there is evidence of misalignment; Extract 3B Kate refers to her guilt and her wish to rid herself of this feeling (turns 6 and 8), whereas the therapist persists in maintaining that Kate’s anger is understandable (turns 7 and 9). This misalignment serves the therapist’s interactional project as she sidesteps the issue of guilt and self-blame and underscores the idea that Kate’s anger is the ‘natural’ response to her father’s behavior. This is met with further resistance, however, as Kate next represents her own inability to accept her father’s behavior as the problem, as shown in Extract 3C.

**EXTRACT 3B:** From 9:36:55 to 9:37:45.

6	K	(4) ((*K bites lips*)) and this, with the guilt
7	Th	Although, yes, of course (.) [you get angry]
		((*T shrugs*))
8	K	Hhh (4) and this, about this guilt, I also try to understand it because it is (.) I don’t want to feel that (.) [no one wants to feel that
9	Th	[you feel guilty] every time you get angry with him ((*K touches her hair, looks down, clears her throat, bites her lips*)) (1) bu:t (2) how could you not be? (3) ((*K bites her lips*)) you describe a father who.hhh ((coughs)) disappoints you (.) I won’t say all the time, but disappoints you often
10	K	(6) Hhhhh
11	Th	In smaller and in more serious ways
		((*T empathic facial expression*))

**EXTRACT 3C:** From 9:37:46 to 9:40:56.

12	K:	((sighs)) (8) I don’t know (7) Hhhhh I think I need to find a way to (.) accept it (1.5) things will not change (.) >my father will not wake up one morning and start sayi:ng well done<, for bigger or smaller things, or <in his own way> show it ((clears throat))
		((*T looks away*))
13	T:	It looks like, though, that right now.hh you cannot accept.hh how much this angers you (.) how much it disappoints you (4) ((*K is about to speak, T speaks before her*)) so, you say, I MUST ACCEPT IT (.) ok, it may be °that you must accept it° but<right now> this is not °what you feel°
		((*K purses her mouth*))
14	K:	(2) I say that I must accept it because it is something that will not change
15	T:	Yes, but right now (.) you (.) need (.) his recognition for what you are, for all the things that you do for him, and when you don’t get it (.) it costs you a lot and you get angry with him (.)
		((*K wrings her hands, nods, looks down*))
16	K	(1.5) And with myself ((*T looks away, shifts leg position*)) I get angry with myself too, as well as getting angry with him
17	T	Yes, and then you feel guilty because you (.) the next day you tell him again ((tsch)) trying to give him an opportunity to repair, HOPING that this time (.) he will say something better, he doesn’t do that your father
18	K	((*K smiles slightly, looks down, plays with her hands, mouths ‘no’*))
19	T	and the disappointment grows (.) you feel like an idiot like you said (3) and of course, you become less tolerant emotionally (.) it is already massive, the tolerance you show °in this situation° and then
20	K	((Sighs))
21	T	when you get angry, you suffer (.)°you feel° (.) °°that you have done something bad°°
		((*K purses her lips, nods slightly, looks down*))
22	K:	(5) ((*K forced smile, like a grimace*)) I’m either angry hehehe or guilty ((forced smiles))
23	T:	(1.5) .hh (.) I think the guilt is there because you °get angry°
24	K:	Yes, that’s why I said it >as soon as the anger passes the guilt starts<
25	T:	It is as if you believe, like ((T shrugs)) that you shouldn’t get angry
26	K:	(2.5) Yes, >I shouldn’t pay him any attention<
27	T:	Or that it would be possible for someone not to get angry ((*K bites her lips*)) (7) Yes (.) you could pay him no attention if ((coughs)) if you didn’t do a thousand things for him
28	K:	(4) ((sighs))

In response to the therapist’s rephrasing formulation, Kate is silent and then shifts the focus back on herself and the need for her to accept how things are (turn 12). In terms of clinical process, it seems that Kate’s resistance intensifies here: instead of recognizing her father’s failings and the feelings these engender in her, she blames herself for the way she feels. The therapist responds with another rephrasing formulation that concerns the here-and-now of the session (turns 13 and 15). In psychoanalytic terms, this is a defense interpretation: Kate’s wish to accept things results from her difficulty in accepting her disappointment in her father and is, therefore, a defense. In response, Kate’s self-blame becomes even more explicit (turn 16) but the therapist ignores this (turns 17, 19, and 21) and restates her formulation. In the final part of this sequence, the therapist introduces another layer in her formulation (turns 25 and 27): the problem lies in Kate’s expectation that she should not get angry and her self-criticism and this leads to Kate eventually get in touch with the sadness (turn 28).

**FIGURE 5:**
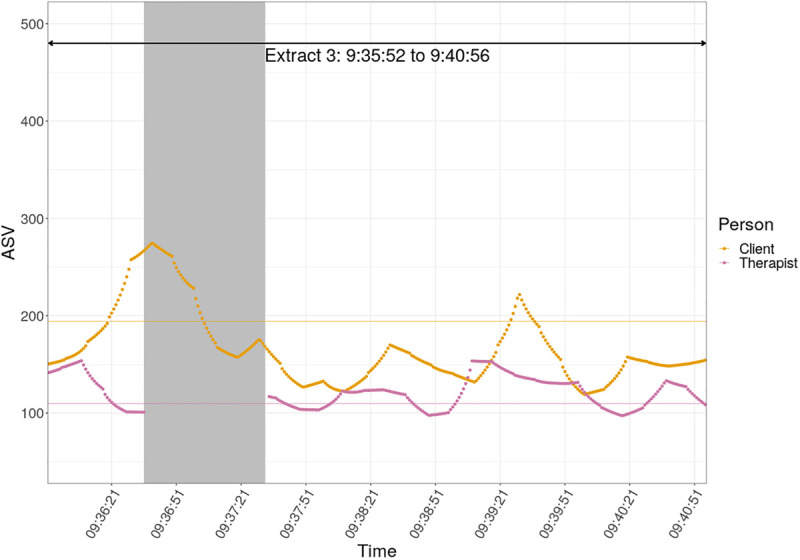
Absolute stress vector against time for client and therapist, TE6.

In terms of autonomic arousal (Figure 5), Kate’s arousal gradually decreases as the interaction unfolds; indeed, Kate’s mean ASV is at its lowest in this part of the session. This is interesting, given that the interaction in this topical episode is intense in terms of clinical work. The therapist’s ASV on the other hand rises when Kate starts to talk about getting angry with herself and during the delivery of her interpretation (turns 16 to 25, from 9:39:09 to 9:40:30).

The interaction described above is quite typical of the therapeutic work in the latter part of the session. Most of the therapist’s turns take the form of rephrasing formulations that concern aspects of Kate’s affective experience, namely her hurt and disappointment, that the therapist considers to lie ‘behind’ her current difficulties, namely her anger, guilt and self-blame. The therapist persistently brings these unacknowledged feelings to the fore, represents them as linked with her more conscious feelings, and highlights her resistance to acknowledging these feelings in the here-and-now of the session through self-blame. The therapist can be seen to gradually build a psychoanalytic interpretation that introduces a different perspective regarding her guilt, thus challenging and expanding Kate’s understanding of her own experience; these challenges are accompanied by displays of affiliation. In this process, the therapist assumes semantic and interactional dominance; she introduces topics and new words in the conversation, and -in some instances- does not offer the floor or her turn overlaps with Kate’s.

## Discussion

This study aimed to combine a detailed description of one session of psychodynamic therapy through the analytic tools provided by conversation analysis, whilst paying close attention to nonverbal interaction, with insights gained from examining the trajectory of autonomic arousal of participants through the session. The starting point for this exploration is a recognition that the process of therapy takes place on both explicit/verbal and implicit/nonverbal/procedural levels (BCPSG, 2008, 2010) and, therefore, that affective nonverbal displays by therapist and client are fundamental to the co-creation of meaning and therapeutic change. Conversation analysis provides many tools for examining talk-in-interaction in psychotherapy, and several interesting insights have been generated through discursive research on psychotherapy (e.g., Peräkylä et al., 2008; Smoliak and Strong, 2018). The implicit or procedural level of interaction, however, although recognized as fundamental to the process of therapy, is harder to grasp with discursive methods. Our interest in noting nonverbal displays when analyzing conversation and, importantly, in including measures of autonomic arousal in our study are in the spirit of exploring ways to include the implicit realm when studying psychotherapy process.

There is an extensive research literature on infant-parent interaction using multimodal microanalysis to examine live embodied interaction (Beebe, 2014, 2017), and it has recently been suggested that this could be extended to the study of implicit processes in psychotherapy (Beebe, 2017). Indeed, some recent studies have applied microanalysis to psychotherapy with interesting results (e.g., Harrison and Beebe, 2018; Avdi and Seikkula, 2019; Avdi et al., 2020; Graver, 2020). Drawing upon the literature on infant-parent interaction may provide conversation analysts with both concepts and analytic tools that can help include the implicit domain when studying the process of therapy.

In this case study, although the analysis was data-driven to a large extent, the interactional processes that were observed were conceptualized through the lens of psychoanalytic theory. Conversation analysis provides psychotherapy researchers with powerful tools to examine in detail the minutiae of therapeutic interaction. We suggest that theorizing such descriptions through specific clinical theories can help bridge the gap between psychotherapy research and clinical practice, and provide clinically relevant descriptions of therapy process, contribute to the development of clinical theory, and promote therapist reflexivity.

In this study we expanded the focus of analysis from brief interactional sequences to longer stretches of talk spanning the whole session in an attempt to track the development of meanings over time (Buchholz and Kächele, 2017). This ‘zooming out’ allowed us to observe what, from a psychoanalytic perspective, would be considered the process of free association. The client in this session, especially in the initial part, often accepted the therapist’s formulations minimally and at times disconfirmed them; the therapist remained silent in response and the client next narrated stories that were thematically relevant to the therapist’s invitation and arguably constitute delayed ‘extensive agreements’ (Bercelli et al., 2008). The therapist’s formulations were thus resisted initially but responded to with a time delay, and presumably non-consciously.

Focusing on longer stretches of talk also allowed us to observe the structure of the session as a whole. As the session progressed, the therapist became markedly more active in interpreting Kate’s resistance and this more challenging work came only after a long period during which the therapist primarily listened and reflected Kate’s underlying feelings. As described in the analysis of the conversation, the therapist shifted from allowing Kate’s associations to emerge with minimal interventions on her part, to more actively countering Kate’s resistance as it emerged in the session. However, the more challenging interpretative work was always accompanied by an affiliative and empathic stance on the part of the therapist. In this sense, the ‘holding’ and the insight-oriented aspects of psychoanalytic work can be seen to be used in conjunction with each other and to reinforce each other (Gabbard and Westen, 2003). Moreover, examining the conversation through the session illustrated repeated cycles between resistance and transient affective insight, which is not uncommon in clinical practice. Analyzing the whole session, rather than focusing on specific moments of change, illuminated the slow and painstaking therapeutic work undertaken in helping clients overcome their defenses against painful affect. This is in line with the psychoanalytic perspective, whereby resistance is not considered a failure in interaction but an opportunity to explore unconscious conflict (Greenson, 1967).

In terms of therapist technique, it was interesting to note that in this session, there were no instances of ‘pure’ extensions, i.e., therapist responses that merely reflect the client’s preceding turn. Even in the briefest of her formulations, the therapist introduced a slight shift, as she tended to orient to unexpressed, i.e., unconscious, affects. This may be specific to psychoanalytic therapy, which attends to potential hidden meaning in the client’s utterances and actions (Greenson, 1967). Another key aspect of this therapist’s technique was the use of nonverbal behavior as an interactional resource. As discussed in the analysis, she tended not to display affiliation toward Kate’s storytelling, particularly in the earlier phases of the session; this could be seen as a way of maintaining affective tension, which would then lead to affective expression and self-reflection (Benecke et al., 2005). Later in the session she delivered her interpretative statements which were far more challenging than her interventions early in the session, at the same time she increased her nonverbal affiliative displays and manner. We believe this maintained the alliance and promoted a sense of safety around the interpretative challenges.

### ASV Data

With respect to autonomic arousal, the observation that the therapist’s arousal is markedly lower than the client’s, and with lower variance over time, points to the differing roles of the two participants and the different intensity with which they engage in the affective work of the session. There is some evidence that therapists’ affect regulation capacities are well-developed through their training and clinical experience (Messina et al., 2013; Soma et al., 2020). As can be seen in Figures 1, 2, as the session progresses, the therapist’s physiological arousal is stable in both intensity and variance. In contrast, there are marked differences in the client’s level of arousal in different topical episodes, with the initial part of the session showing both higher arousal and high variance in ASV, which could be seen to reflect shifts in affective state. It seems that for the client, the initial part of the session, during which she narrates several ‘problem’ stories and the therapist gently reflects the underlying sadness and disappointment, is associated with more autonomic arousal and more intense affective shifts, in comparison to the latter part of the session. This is interesting, given that as the session progressed, and in particular TEs 6 and 8, entail more intense interpretative work and arguably more challenge. One hypothesis could be that, following the expression of anger and frustration in the first part of the session, Kate later begins to experience feelings of sadness, associated with lower autonomic arousal (Kreibig, 2010). Another hypothesis could be that the reduced arousal in the latter part of the session is not about the specific affect but may follow reduction in Kate’s internal conflict. This is in line with observations that suppressing emotional expression may be associated with increased arousal (Perrone et al., 2014). In addition, it was interesting to observe that in the early phases of the session there are several points at which the therapist’s ASV became elevated in response to Kate’s storytelling, although there were no visible markers of this arousal.

### Overview

Although only a single session of a single case, we believe that the findings support somewhat extending and expanding the foci of conversation analytic research on psychotherapy. One aspects of the extension in this study was temporal: moving from the traditional CA focus on specific speech turns to the entire time span of the session. This was not a single shift but a process of repeated zooming out and back in. Expanding the focus was threefold. The first expansion, to focus not just on talk is of course not new in CA, but we believe the detailed attention to the prosody and nonverbal information from the session video helped develop earlier analyses that were primarily based on the session transcript. The second expansion has been to draw explicitly on psychoanalytic and infant development theories in contrast with CA’s more traditional quasi-atheoretical approach to analysis. The final expansion has been to draw on the ASV data, through simple, largely visual, inspection of the ASV against the analysis of conversation. This, we believe, showed interesting patterns, including the therapist’s short periods of increased ASV early in the session not associated with any visible markers of arousal, the trend of decreasing physiological arousal over the session for the client, and associations of ASV with talk at the extract level. We encourage others to explore the possible gains from including nonverbal displays and adding physiological information to detailed analysis of talk, in the attempt to learn more about what actually happens in the therapeutic consulting room.

## Data Availability Statement

The raw data supporting the conclusions of this article will be made available by the authors, without undue reservation.

## Ethics Statement

The studies involving human participants were reviewed and approved by Scientific Board, Hellenic Centre of Mental Health and Research. The patients/participants provided their written informed consent to participate in this study. Written informed consent was obtained from the individual(s) for the publication of any potentially identifiable images or data included in this article.

## Author Contributions

EA and CE contributed to the conception and design of the study. EA contributed to acquisition of data, analysis of conversation, and reviewing literature. CE contributed with discussion of the CA through its development. CE contributed to quantitative analyses and plots. EA drafted the manuscript. EA and CE revised the manuscript. Both authors contributed to the article and approved the submitted version.

## Conflict of Interest

The authors declare that the research was conducted in the absence of any commercial or financial relationships that could be construed as a potential conflict of interest.

## Footnotes

1 For a list of publications using Firstbeat measurement devices see: https://www.firstbeat.com/en/science-and-physiology/white-papers-and-publications/.
